# Validation of a Geriatric Bedside Swallowing Screen (GEBS): Protocol of a Prospective Cohort Study

**DOI:** 10.2196/46252

**Published:** 2023-08-11

**Authors:** Susanne Maria Javorszky, Raphael Reiter, Bernhard Iglseder

**Affiliations:** 1 Institute for Nursing Science and Practice Paracelsus Medical University Salzburg Austria; 2 Department of Health Sciences University of Applied Sciences Vienna Austria; 3 Department of Geriatric Medicine Christian Doppler University Hospital Paracelsus Medical University Salzburg Austria

**Keywords:** dysphagia, geriatrics, swallowing disorder, assessment, screening, cohort study, multimorbidity, hospital setting

## Abstract

**Background:**

Demographic changes will raise the need for specialized care of older patients. Oropharyngeal dysphagia has recently been declared a geriatric syndrome reflecting its multifactorial background. Alongside multimorbidity, sarcopenia, frailty, and disability, swallowing disorders increase with advancing age, with prevalence rates reported to be as high as 44% in acute geriatric hospital settings and 80% in long-term care facilities. Hence, systematic screening of older patients to diagnose dysphagia and initiate treatment is of paramount importance to prevent bolus death, aspiration pneumonia, and malnutrition and improve quality of life. Several screening tools have been evaluated in emergency and stroke units. However, no published dysphagia screening tool has been validated in the hospitalized, older adult population using a gold standard in dysphagia diagnostics as a reference test. The validation of the proposed test is a first step.

**Objective:**

The Geriatric Bedside Swallowing Screen (GEBS) study aims to validate a new screening tool developed specifically for older inpatients against an instrumental swallowing evaluation, the flexible endoscopic evaluation of swallowing (FEES), which is considered a gold standard. Primary outcomes to be evaluated are sensitivity and specificity for the GEBS in the detection of dysphagia in a mixed older adult population. The presence of dysphagia will be defined by an instrumental swallowing evaluation (FEES), analyzed by the standardized penetration-aspiration scale.

**Methods:**

To validate the GEBS, a prospective cohort study will be carried out. Two institutions, an acute geriatric department and a long-term care facility, will aim to recruit a total of 100 patients aged ≥75 years. After giving their informed consent, patients will undergo the full screening protocol described in the GEBS as well as an evaluation of swallowing function using the FEES. Investigators will be blinded to the results of the respective other testing. The analysis of pseudonymized data sets will be done by a third investigator. Outcomes to be considered are sensitivity, specificity, diagnostic odds ratio, positive and negative likelihood quotient, and the reliability of the proposed dysphagia screening tool using the κ coefficient.

**Results:**

Recruitment started in October 2022 and will end in April 2024. Data publication is planned for early 2025.

**Conclusions:**

If proven to be a valid screening tool for the early detection of dysphagia, further studies including different older adult populations as well as studies to determine the impact of systematic dysphagia screening on parameters, such as rates of aspiration pneumonia or nutritional status, should be planned. Effective screening of dysphagia will lead to earlier detection of patients with impaired swallowing. Those who fail the screening will be referred to speech language pathology for further diagnosis, thus optimizing care while streamlining personnel resources.

**Trial Registration:**

ISCRTN Registry ISRCTN11581931; https://www.isrctn.com/ISRCTN11581931

**International Registered Report Identifier (IRRID):**

DERR1-10.2196/46252

## Introduction

### Background

Due to demographic changes, by 2100, about a third of the population in the European Union will be aged 65 years and older [1]. Medical professions will need to be specialized in the treatment of older patients. This includes the development of efficient diagnostic pathways to ensure optimal care while not overburdening health care systems. Thus, efficient prevention, early detection, and treatment are becoming more important. One important discipline in the care for older patients is *speech language pathology* (SLP), which contributes to the prevention, diagnosis, and treatment of hearing disorders; voice impairments; difficulties with language production; active communication; and swallowing disorders, also known as *dysphagia*.

Dysphagia has been declared a geriatric syndrome by the European Society for Swallowing Disorders, thus highlighting its importance for medical personnel working with older patients. Common causes for dysphagia are stroke, traumatic brain injury, neurodegenerative diseases, as well as surgeries—most commonly following cancer treatment of the mouth and neck. It is important to note that changes to the swallowing process due to aging, which is known as *presbyphagia,* are not considered pathological. If presbyphagia does not cause nutritional decline or the development of aspiration pneumonia, presbyphagia is considered healthy, older-age swallowing [2,3]. If the aging process, specifically the loss of muscle mass within the swallowing muscles, leads to functional deficits and disordered swallowing with pathological consequences without a known neurological or structural cause, it is defined as *sarcopenic dysphagia* [4]. To distinguish between presbyphagia and dysphagia, regardless of etiology, sound knowledge of a healthy, older-age swallowing process is necessary. The loss of muscle mass associated with normal aging, that is, *sarcopenia*, causes *dynapenia,* which is a loss of function and strength within the skeletal muscles [5]. Continual sarcopenia leading to functional decline contributes to the geriatric syndrome known as *frailty,* which is linked to a higher prevalence and worse prognosis for dysphagia [6]. Dysphagia of all different etiologies affects up to 40% of patients in acute hospital settings as well as up to 80% in long-term care facilities [7-9]. The prevalence of sarcopenic dysphagia in older patients admitted to acute hospitals has been determined to be around 30% [10]. As oropharyngeal dysphagia is closely associated with central nervous system diseases, the prevalence for dysphagia in patients with central nervous system diseases is higher as the diseases progress, with estimates of around 85% in older patients [9].

The diagnosis of dysphagia is typically a 2-step process with a clinical swallowing examination by an SLP, who might refer to an instrumental swallowing examination. There are 2 objective instrumental diagnostic tools. The *flexible endoscopic evaluation of swallowing* (FEES) consists of a protocol to evaluate different consistencies of bolus under visual observation of the pharynx and upper laryngeal structures through a transnasal endoscope [11]. The second possibility is a *videofluoroscopic swallowing study*, where a high-resolution x-ray of the mouth and throat produces a video of the whole swallowing process [12]. Since the FEES system is portable, it can be carried out in a bedside setting and does not require as much compliance as the videofluoroscopic swallowing study. The FEES is considered the gold standard in the dysphagia diagnostics of older patients [13].

### Study Goal

As previously described, the identification of patients with impaired swallowing function is of high priority to prevent the occurrence of malnutrition and aspiration pneumonia, as well as bolus death. Considering the demographic changes, routine referral of all newly admitted older patients to an SLP examination will not be feasible. Screening for dysphagia risk has proven to significantly lower pneumonia rates in emergency and stroke units [14-17]. There are several validated screening tools for those populations, for example, the *Gugging Swallowing Screen* [18] or the *Toronto Bedside Swallowing Screening Test* [17], which have excellent sensitivity and specificity for their intended use. Older patients, however, are faced with the challenge of drastically increased time frames for the oral phase of swallowing. This would be a predicator for dysphagia in healthy, younger adults but does not have the same implication in older adults [19].

To search for a screening tool that is validated for an older adult population aged ≥75 years against a gold standard, a systematic review of published screening tools was carried out by the first author, which yielded no results [20]. Of the 40 screening tools that were found (as shown in Table 1), none could be included for further assessment, with the main exclusion criteria being a noninstrumental reference test and a studied population aged <75 years. Some of the studies proposed a new protocol of instrumental swallowing assessments or a clinical bedside swallowing evaluation by an SLP under the term “screening”; those studies were also excluded.

These findings have been replicated by a recent review by Estupiñán Artiles et al [21], as well as the *Arbeitsgemeinschaft der Wissenschaftlichen Medizinischen Fachgesellschaften* (Association of the Scientific Medical Societies in Germany) guidelines for geriatric assessment [22].

**Table 1:** Review of published screening tools.

Exclusion criteria	Screening tools (n=40), n (%)
Reference test	1 (2.5)
Population	8 (20)
Reference test + population	26 (65)
Reference test + population + screening tool	2 (5)
Screening tool + population	3 (7.5)

In accordance with diagnostic study guidelines such as STARD (Standards for Reporting Diagnostic Accuracy) and QUADAS (Quality Assessment of Diagnostic Accuracy Studies), new tests should be validated against a robust reference test, usually the gold standard [23,24]. Following these outcomes, this study aims to fill the gap by validating a specific screening tool—the *Geriatric Bedside Swallowing Screen* (GEBS)—to detect dysphagia in a mixed older adult population aged ≥75 years. The aim is to validate the GEBS using the primary outcome measures of sensitivity and specificity concerning the detection of dysphagia when compared to an instrumental swallowing evaluation.

## Methods

### Test Construction

The starting point for the GEBS was a screening tool already in use at the Department of Geriatric Medicine, Christian Doppler University Hospital, in Salzburg, Austria. It has been demonstrated to be a convenient tool in clinical practice but has never been formally validated. Combined with the results of a thorough review of published evidence on possible reliable predictors for dysphagia, it was developed into a 3-step screening tool. In the first step, 6 risk factors are assessed by observation, patient reports, and chart review (Textbox 1).

Following the assessment of present risk factors and the assurance of patient vigilance, a water swallow test is administered. This consists of swallowing 10 teaspoons of clear water with breaks for voice production and 1 self-administered sip out of a cup. The test is stopped if clinical signs of aspiration such as coughing or wet voice are noted, as well as after the occurrence of one of the following items is noted: breathing difficulties, as a result of the lack of maintaining alertness, head position, or saliva control.

If the water test shows signs of dysphagia and more than one risk factor is present, the third step will be administered, which consists of swallowing a pureed bolus with a consistency of level 4 in the *International Dysphagia Diet Standardisation Initiative* (IDDSI) framework [25]. After making sure that the patient is able to stay awake and upright for 15 minutes; has control over saliva, for example, shows no sign of drooling; and is able to produce voice, several teaspoons of puree are administered with breaks for voice production. The screening must be stopped for decreased vigilance, clinical signs of aspiration, and severe drooling without compensation. Coughing, wet voice, and oral residues are considered indicators for dysphagia.

A point score will be calculated, where a score of 5 points would allow for oral nutrition and medication with pureed food of IDDSI level 4 and thickened liquids of IDDSI level 2 until further examination by an SLP; a score of <5 points would result in the instruction of *nil by mouth* until further examination with a high risk for dysphagia.

This 3-step process of determining risk factors; applying a water-swallow test; and if necessary, administrating a pureed bolus test ensures patient safety while aiming for a high specificity and sensitivity [21,26].

**Risk factors for dysphagia.**

1. Neurological or neuropsychiatric disease
2. Pneumonia within the last 12 months
3. Sarcopenia
4. Unintentional weight loss
5. Symptoms of dysphagia (drooling, wet voice, and coughing)
6. Impaired oral health

### Recruitment

Two Austrian institutions will recruit a total of 100 patients with and without known or suspected dysphagia. One study center is an acute geriatric department, whereas the other is a long-term-care facility. Patients’ inclusion criteria are an age of 75 years or older and the ability to give informed consent. Exclusion criteria are a history of severe brain stroke with a cut-off of 20 points on the *National Institutes of Health Stroke Scale* [27]; a diagnosis of moderate to advanced stages of cognitive diseases defined by 20 points or lower on the *Mini Mental State Examination* [28]; as well as the presence of a tracheostoma with or without dependence on ventilation [29]. Independently of age, these 3 factors are associated with a high prevalence of dysphagia and would be considered too high for risk of bias for the first validation on a mixed older adult population. All patients admitted to the acute geriatric department after the start of recruitment fulfilling the inclusion criteria will be considered and asked to participate in the study. In the long-term-care facility, staff will screen patient data and nominate possible participants, who will then be approached for their consent to participate. Recruitment started in October 2022 with a planned time frame of 18 months.

### Application of the Index Test GEBS

Patients who have agreed to participate in the study will be screened with the full GEBS even if they do not show a risk of dysphagia after the first 2 steps. If any occurrence during the screening indicates dysphagia and requires the screening to be stopped and the patient to be referred to further assessment, the test result can be included in the study as “fail.” To ensure broad validation of the GEBS, the screening can be carried out by nurses, physicians, or SLPs. Investigators will be educated on the correct process and scoring of the GEBS prior to the study. To ensure intrarater reliability, 15 patients will be screened twice with the GEBS by the same investigator with a minimum of 4 hours between both events. To calculate the interrater reliability, another 15 patients will be screened twice by 2 independent investigators blinded to the respective other result of the GEBS.

### Application of the Reference Test FEES

Within 7 days of the first assessment using the GEBS, the reference test FEES will be carried out to verify the results. This time frame limits possible changes in the patients’ medical status between the 2 investigations of their swallowing function. The FEES is considered the gold standard, objective diagnostic tool for dysphagia in older patients and is considered particularly safe and only mildly uncomfortable for the majority of patients. Reported adverse events are epistaxis, bradycardia or tachycardia, lowering of blood pressure, and changes in heart rate, all of which are rare with a reported occurrence of less than 4%. None of these reported events that were directly caused by undergoing the FEES have ever been reported as leading to permanent damage; all are self-limited and of a very short duration [30,31]. The results of the FEES will be reported using the *penetration-aspiration scale* developed by Rosenbek et al [32] in 1996; it is an internationally established 8-point scale to rate the severity of dysphagia. To limit uncomfortable sensation for the patients while the endoscope is passed through the nasal cavities, a local anesthetic can be used, which does not affect the swallowing function if used correctly [33,34]. The FEES investigators will be blinded to the results of the GEBS.

### Statistical Analysis

#### Outcomes

The 2 main outcomes to be analyzed are sensitivity and specificity to detect dysphagia. These will be calculated using a 2 × 2 matrix. Further, positive and negative likelihood quotient and diagnostic odds ratio will be calculated. To assess intra- and interrater reliability, the κ coefficient will be evaluated. Additional cross-analysis with acquired data such as age; gender; medical diagnosis, including severity if assessed (National Institutes of Health Stroke Scale and Mini Mental State Examination); current diet; and dependency on activities of daily living will be considered as secondary outcomes. Calculations will be carried out using Excel (Microsoft) and SPSS (IBM Corp).

#### Data Management

Acquired data for the study will be managed and pseudonymized with the keys being stored at the recruiting institution and not available to the study coordinator. Data will be stored using Excel (Microsoft) following the legal requirements of the Austrian government.

### Ethics Approval

Ethics approval has been obtained for both study centers. First, the ethics committee of the City of Vienna approved the study (EK 21-212-1021) in March 2022. The ethics committee of the Province of Salzburg gave approval (EK-Nr. 1040/2022) in June 2022. Required changes were the complete exclusion of patients who are not able to give their informed consent due to cognitive diseases as well as minor clarifications in the patients’ information document. The study was registered with the ISCRTN registry (11581931) in March 2021.

## Results

Recruitment started in October 2022 and will end in April 2024. A planned total of 100 participants will be aimed for. Since there have not been prior studies using the GEBS, no expected effect can be used to calculate a power analysis. Regarding previously published, internationally established screening tools, a case number of 100 patients was decided on. However, if, by the end of the study period, fewer results have been obtained, recruitment will not be extended further. Data publication is planned for early 2025, following the end of the collection period.

## Discussion

### Expected Findings

The GEBS is, to our best knowledge, the first screening tool for the assessment of dysphagia in older patients to be validated against an instrumental swallowing assessment in accordance with guidelines, such as STARD and QUADAS, while taking into consideration individual predisposing factors. This is highly relevant considering the growing demand for personalized medicine in this age group. Similar to children not being small adults in regard to medication, older patients with frailty are to be considered differently for their changed muscle function, metabolism, and physical activity.

This study aims to provide geriatric facilities in the acute setting as well as in long-term care with a reliable, useful tool to screen patients for dysphagia. If the GEBS shows sufficient results to be recommended for broader use in different geriatric settings, further studies in different geriatric settings are advisable. The impact of systematic dysphagia screening with the GEBS should be evaluated by using end points such as rates of aspiration pneumonia, nutritional status, occurrence of bolus death, as well as the patient’s quality of life. Other populations such as patients with moderate to severe cognitive decline or tracheotomy tubes should also be included in further studies, to determine the validity of the GEBS in different patient groups.

Several studies have shown significant changes in the parameters mentioned above, namely pneumonia rates, nutritional status, bolus death, and quality of life, upon systematic screening for dysphagia in emergency departments or stroke units, so there is reason to expect similar effects in geriatric facilities when using a specialized geriatric dysphagia screening tool [14,16].

The screen will be used as a 2-step process in clinical use after having been validated, so the third part will only be administered if no SLP is available. If patients show a risk for dysphagia during the water swallow test and an SLP is available, the screen will be discontinued, and the patient will be referred to an SLP evaluation straight away. Especially in long-term care facilities in Austria, the presence of an SLP is not always the case. Thus, it is necessary for nursing and medical staff to be able to determine the safest method of oral intake until the swallowing function can be assessed by a specialist.

To spare patients unnecessary diagnostic processes and restrictions in their oral intake, specific diagnostic pathways and case-specific treatment need to be administered. A fast and safe way to ensure oral intake and medication in older patients can assist doctors and nursing staff upon the admission of new patients and streamlines SLP personnel resources.

### Limitations

A limiting factor is the exclusion of patients with moderate to severe cognitive diseases. This is a growing population that is highly susceptible to developing dysphagia. This might be a future project after a first successful validation of the GEBS.

### Conclusions

With this study, we hope to fill the gap in the health care of the older adult population by minimizing risk factors for patients and workload for caregivers at the same time. Optimized protocols and diagnostic pathways can be developed by implementing systematic, low-level screening protocols. If the results are positive in terms of high sensitivity and specificity, the GEBS will be published internationally, which might have a positive impact on workplaces and the health care of older patients.

## Abbreviations

FEES: fiberoptic endoscopic evaluation of swallowing
GEBS: geriatric bedside swallowing screen
IDDSI: International Dysphagia Diet Standardisation Initiative
SLP: speech language pathology (or pathologist)
STARD: Standards for Reporting Diagnostic Accuracy
QUADAS: Quality Assessment of Diagnostic Accuracy Studies
